# Analysis of the TREC and KREC Levels in the Dried Blood Spots of Healthy Newborns with Different Gestational Ages and Weights

**DOI:** 10.32607/actanaturae.11501

**Published:** 2022

**Authors:** D. A. Cheremokhin, K. Shinwari, S. S. Deryabina, M. A. Bolkov, I. A. Tuzankina, D. A. Kudlay

**Affiliations:** Institute of Immunology and Physiology of the Ural Branch of the Russian Academy of Sciences, Yekaterinburg, 620049 Russia; Medical Center “Healthcare of mother and child”, Yekaterinburg, 620041 Russia; Department of Immunochemistry, Institute of Chemical Engineering of the Ural Federal University, Yekaterinburg, 620083 Russia; I.M. Sechenov First Moscow State Medical University (Sechenov University), Moscow, 119991 Russia; National Research Center, Institute of Immunology Federal Medical-Biological Agency of Russia, Moscow, 115522 Russia

**Keywords:** T-cell receptor excision circles, K-deleting recombination excision circles, primary immunodeficiency, inborn error of immunity, severe combined immunodeficiency, reference value

## Abstract

Inborn errors of immunity can be detected by evaluating circular DNA (cDNA) fragments of T- and B-cell receptors (TREC and KREC) resulting from the receptor gene rearrangement in T and B cells. Maturation and activation of the fetal immune system is known to proceed gradually according to the gestational age, which highlights the importance of the immune status in premature infants at different gestational ages. In this article, we evaluated TREC and KREC levels in infants of various gestational ages by real-time PCR with taking into account the newborn’s weight and sex. The 95% confidence intervals for TREC and KREC levels (expressed in the number of cDNA copies per 105 cells) were established for different gestational groups. The importance of studying immune system development in newborns is informed by the discovered dependence of the level of naive markers on the gestational stage in the early neonatal period.

## INTRODUCTION

Innate errors of immunity (IEI), which are also known as primary immunodeficiency (PID), are a group of genetic diseases that manifest themselves as various developmental defects and immune system dysfunction. In 2019, the International Union of Immunological Societies classified and listed more than 450 individual IEI [1]. Thanks to advances in our understanding of their pathogenetic basis and improvement in laboratory diagnostic methods, it has become possible to provide a large number of patients with a clinical diagnosis confirmed by the results of molecular genetic studies. The IEI prevalence currently stands at 1.27 per 10,000 cases [2, 3].

V(D)J recombination is one of the most important events taking place in a functional immune system, during which diverse and functional variants of T-and B-cell receptors (TCR and BCR, respectively) and antibodies are formed. These processes are essential stages in adaptive immunity development [4]. The recombinases RAG1 and RAG2 play an important role in this process [5]. These proteins catalyze the rearrangement of the DNA fragments of TCR genes during T cell maturation and the B cell response at the stage of selection of variable regions of immunoglobulins.

T-cell receptor excision circles (TREC) are DNA fragments resulting from the TCR gene rearrangement in thymocytes. TREC are transported as episomal DNA from the nucleus to the cytoplasm of independent, although still naive, T cells, where they persist without being involved in replication during mitosis. The resulting TREC concentration indicates the number of naive T cells, which is, apparently, an important diagnostic criterion [6, 7, 8]. Double-stranded DNA circles similar to TREC are formed during BCR gene rearrangement in naive B cells; they are called kappa-deleting recombination excision circles (KREC) [9]. KREC resulting from intron RSS–Kde rearrangement at the IGK locus is used to assess B-cell neogenesis in the bone marrow [10, 11]. Both TREC and KREC are non-replicative and stable; their levels do not change during cell proliferation (e.g., clonal expansion) [12, 13]. Because of that, quantification of TREC and KREC molecules is widely used to assess the state of the thymus and bone marrow in various physiological and pathological conditions. The blood levels of TREC and KREC can be a criterion of high diagnostic significance in various immunodeficient states. A method for multiplex real-time PCR that makes it possible to detect defects in T and B cells generation by simultaneously measuring TREC and KREC copy numbers has been developed [14, 15]. Mass screening for the TREC/KREC level could help us classify an infant as a risk group patient based on their immunological profile as early as in the neonatal period; this will increase the survival rate of infants with an immune-related pathology and reduce expenses [16, 17, 18]. This approach has other advantages, including high sensitivity, high throughput capacity, relatively low cost, and the possibility of using DNA isolated from the minimum volume of a blood sample collected using Guthrie cards [6, 16, 19]. This allows for using TREC and KREC molecules as functional markers of the thymus and bone marrow in various clinical conditions and, in particular, IEI. However, in order to characterize IEI patients, one has to know the state of these immunity markers in a healthy individual, especially with taking into account his/her age and sex [20]. Quantification of TREC and KREC in a newborn’s blood remains topical.

## EXPERIMENTAL

**Dried blood spot samples**

Our study included 80 dried blood spot (DBS) samples obtained from otherwise healthy (no deviations according to the results of large neonatal screening and blood transfusion data) infants (40 boys and 40 girls) collected onto Perkin Elmer 226 Guthrie cards (Perkin Elmer Health Sciences, USA). The Guthrie cards were stored at the Neonatal Screening Laboratory of the Medical Center "Healthcare of mother and child" (Yekaterinburg) at room temperature before use.

**DNA isolation**

DNA was isolated from seven DBS discs 3.2 mm in diameter (20 µl) by magnetic sorting on the Magna Pure LC 2.0 Instrument using the MagNA Pure LC DNA Isolation Kit I (Roche Diagnostics GmbH, Germany) according to the standard DNA I Blood_ Cells_High_Performance protocol. The DBS pretreatment step included sample lysis using the buffer from the Magna Pure LC DNA Isolation Kit II (Tissue). A total of 260 µl of the lysis buffer and 40 µl of proteinase K were added to the DBS samples. The resulting mixtures were thoroughly vortexed and incubated at 65°C for 20 min with occasional tube shaking and, then, at 95°C for 10 min with shaking on a vortex every five min. The samples were cooled to room temperature, and the extract was transferred to the Sample Cartridge and loaded into the workstation.

**PCR analysis of TREC and KREC**

The TREC and KREC molecules were quantified by PCR with real-time detection of the fluorescent signal. The study was conducted on a CFX96 qPCR Detection System (Bio-Rad, USA) using the Immuno-BiT reagent kit (ABV-test, Russia) according to the manufacturer’s instructions. The number of TREC and KREC molecules per 105 nucleated cells (leukocytes) was calculated relative to the ALB gene copy number using the following formula:

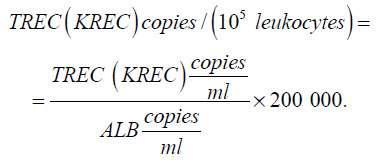



In the case of an ALB copy number < 10^5^, the result was considered invalid, and the study was repeated, starting from the DNA isolation stage.

**Statistical data analysis**

The data were analyzed mathematically using the Microsoft Excel (Microsoft Office 365, USA) and IBM SPSS Statistics V21.0 (IBM Corp., USA) statistical software packages. The normality of the data distribution was assessed using the Shapiro–Wilk test; the arithmetic mean and standard error of the mean (m ± SEM) were used for descriptive characterization. Student’s t-test for independent samples and Pearson’s correlation coefficient (p) were used to analyze the statistical significance of the differences between the mean values and the presence of correlations, respectively. Differences were considered significant at p < 0.05.

**Fig. 1:**
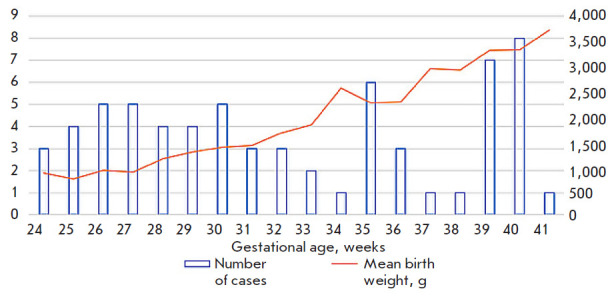
Distribution of healthy infants based on gestational age and birth weight

The study included 80 apparently healthy infants (40 boys and 40 girls) of different gestational ages born in the Sverdlovsk region in 2020 (Fig. 1).

## RESULTS

**Gender differences in the TREC and KREC levels**

**Fig. 2:**
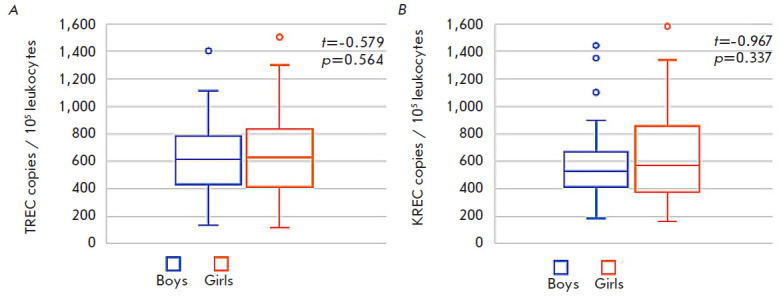
TREC (*A*) and KREC (*B*) levels in the DBS samples of healthy infants depending on gender. *Note: *hereinafter, the median, interquartile, and maximum range are shown

An analysis of the TREC and KREC levels in the DBS samples of the presumably healthy boys and girls revealed no statistically significant differences (Fig. 2), which corresponds to the results obtained by other researchers [21].

**TREC and KREC levels in preterm infants of different gestational ages**

**Fig. 3:**
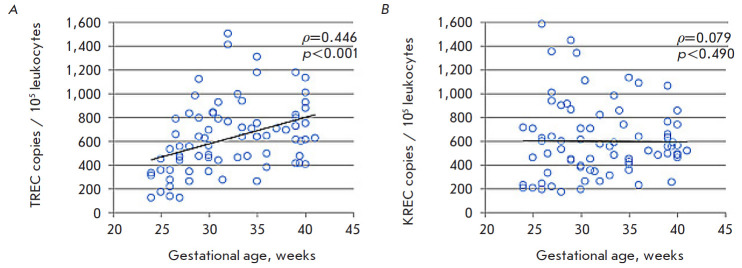
TREC (*A*) and KREC (*B*) levels in the DBS samples of healthy infants depending on gestational age in the early neonatal period

A statistical analysis based on correlation data was conducted to determine possible variations in the TREC and KREC copy numbers associated with a newborn’s gestational age. A statistically significant positive correlation (*ρ *= 0.446 (p < 0.001)) was established between the gestational age and the TREC level. No relationship was found between an infant’s age and the level of KREC, which is a marker of naive B cells (Fig. 3).

**TREC and KREC levels in preterm infants with different birth weight**

**Fig. 4:**
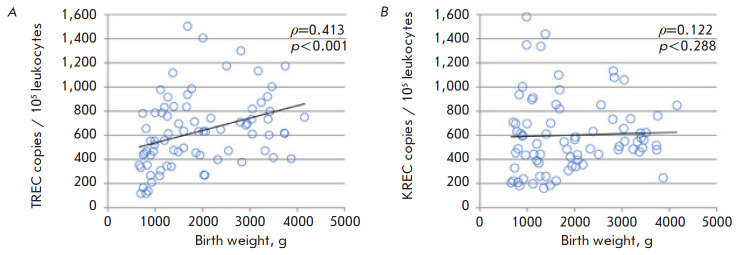
TREC (*A*) and KREC (*B*) levels in the DBS samples of healthy infants depending on birth weight

The relationship between naive T- and B-cell marker levels and a newborn’s weight was also analyzed. Naturally, the fetus develops during pregnancy. All internal organs and, in particular, the thymus, develop according to the gestational period. During their growth and differentiation, thymogenic tissues are enriched with lymphocyte precursors and, in particular with ones that have already passed double recognition; i.e., positive and negative selection. This is denoted by a positive correlation (*ρ *= 0.413 (p < 0.001)) between an infant’s birth weight and the TREC level in their DBS sample. No such correlation was found between the birth weight and KREC level (Fig. 4).

**Analysis of TREC and KREC levels in infants of different preterm birth categories**

**Fig. 5:**
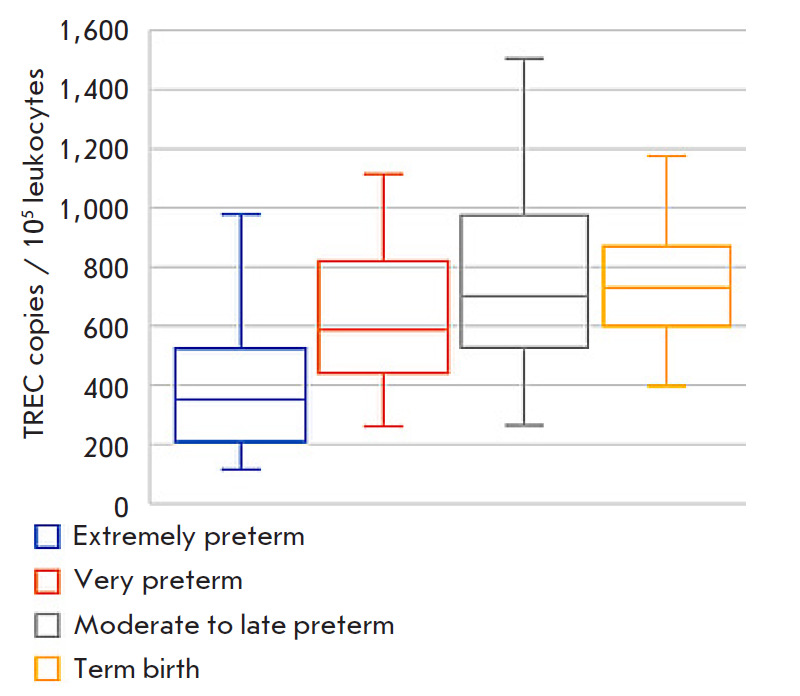
TREC levels in the DBS samples of infants of different gestational ages in the early neonatal period

Having discovered a positive correlation between the newborn’s gestational age and TREC level, we decided to evaluate the possibility of significant differences in the TREC level of infants of different preterm birth categories. In order to do this, we decided to divide newborns into four groups according to the preterm birth category: extremely preterm ( < 28 weeks), very preterm (28–32 weeks), moderate to late preterm (33–38 weeks), and term-birth (39–41 weeks) infants (Fig. 5, Table).

**Table:** TREC and KREC levels (copies/105 leukocytes) in the DBS samples of apparently healthy infants of different gestational ages in the early neonatal period

Preterm birth category	Mean	95% confidence interval	SEM	MIN	MAX
TREC					
Extremely preterm ( < 28 weeks)	402.7	116.6–784.0	52.8	115.9	978.0
Very preterm (28–32 weeks)	611.1	271.0–917.9	52.5	261.4	1115.6
Moderate to late preterm (33–38 weeks)	776.1	378.2–1405.5	76.5	263.6	1505.6
Term birth (39–41 weeks)	723.9	406.1–1133.2	52.2	398.1	1174.0
KREC					
	599.9	210.9–1103.5	34.9	162.8	1584.0

Figure 1 shows a trend towards an increase in the TREC marker level during fetus growth and development; however, there was no statistically significant correlation between the preterm category and TREC level. Since no statistically significant relationship was found between the KREC copy number in the DBS sample and such parameters as a newborn’s body weight and gestational age in infants of different preterm categories, the newborns were not divided into separate groups. An average of 599.9 KREC copies per 105 leukocytes (SE = 34.9) were found per sample; however, one needs to know the lower limits of the obtained reference intervals for practical use. The lowest KREC value in the group of apparently healthy infants was 162.8 copies/105 leukocytes, while the lower limit of the 95% confidence interval was 210.9 copies/105 leukocytes (the results of descriptive statistics are presented in the Table).

## DISCUSSION

To date, there have been numerous attempts at TREC/KREC quantification in different age groups, ranging from infants and older children to adult populations. The main goal of most of these studies was to assess the changes in the functional activity of naive immunity during organism maturation and aging [21, 22, 23]. According to Douek et al., the TREC and KREC copy numbers in the blood samples of older children and adults are 10 and 100 times lower than those in healthy term-birth newborns, respectively. These data indicate a reduced thymic output owing to the age-related decrease in the functional thymus tissue [24]. The study of patients with combined variable immunodeficiency and healthy control donors revealed a stable KREC level, while TREC levels reduced with age in both patients with an immune-related pathology and the control group; this confirms the independence of the decrease in the level of naive T-cell markers from the disease and dependence on age [23, 25, 26, 27]. The TREC and KREC levels may also decrease because of dilution. Nuclear DNA replicates only during an active immune response at the stage of cellular expansion of functionally active lymphocyte clones. Since TREC and KREC are episomal molecules, they remain only in precursor cells, which leads to a relative decrease in the parameters [28]. Despite numerous data on age-related changes in T- and B-cell immunity, it is also important to study TREC and KREC level alternations not only in the early neonatal period, but at different fetal development stages as well. This will allow for assessing the immune state at different stages of human embryonic development.

The main goal of our study was to determine the TREC and KREC levels in the blood of newborns delivered at different gestational ages. However, for greater informational value, we decided to analyze gender differences in the levels of naive immunocompetent cell markers. We have not found any significant differences in the TREC and KREC levels between boys and girls, which is consistent with the results obtained by other researchers [29, 30]. However, some works indicate a higher TREC level in the blood of females [31, 32, 33].

To date, data have been published on the relationship between an infant’s birth weight and his/her TREC level [34, 35, 36]. A recent study of DBS samples of preterm infants revealed a positive correlation between the TREC and KREC levels and birth weight [5]. Another work studied the relationship not only between birth weight, but also gestational age at the time of birth. Based on the analysis results in that work, newborns were divided into three groups: infants with very low, low, and normal birth weight [32]. Data have also been published on the existence of a relationship between the immune state and growth of adult individuals [36, 37].

All internal organs, in particular, the thymus, develop in accordance with the gestational period. During growth and differentiation, thymogenic tissues are enriched with lymphocyte precursors and, in particular, with the ones that have already passed double recognition: i.e., positive and negative selection. This is indicated by a positive correlation (*ρ *= 0.413 (p < 0.001)) between the infant’s birth weight and the TREC level in his/her DBS sample. [21, 26, 27].

We also analyzed the relationship between the quantitative content of B-cell markers, gestational age, and newborn’s weight; however, the relationship between these parameters was not statistically significant. These results show that the KREC level in the DBS of a newborn delivered at a gestational age ≥ 28 weeks, which corresponds to normative values, is an indicator of proper B cell maturation in the bone marrow. Pre-B-cells are found in the fetal liver at week eight of pregnancy, where they already express class M immunoglobulins on their surface by that period. This indicates that the system of humoral parameters of immunity functions with high efficiency by that early stages of human development, which probably determines the relatively high KREC level in the DBS of newborns delivered at different gestational ages [23, 27].

IEI usually remain undiagnosed until clinical signs begin to appear. These signs are, mainly, chronic and recurrent infections. In order to diagnose PID, a clinical blood analysis, lymphocyte phenotyping, determination of various types of immunoglobulins, functional tests for determining immunocompetent cells, and a genetic analysis are used. Functional tests for determining immunocompetent cells and a genetic analysis are currently the most promising and important approaches. The analysis of the TREC and KREC levels is a fast and sensitive tool for screening for severe combined immunodeficiency (SCID) and diagnosing other IEI, especially considering the fact that a small sample volume is required for DNA extraction, which reduces the risk of harm to the infant. The impossibility of collecting the required amount of biological sample is often an insurmountable barrier in laboratory diagnostics. The use of different protocols for TREC and KREC level analysis in different laboratories contributes to the wide spread of thresholds for newborn screening programs among different countries. Genetic differences between populations may also play a role. Therefore, our results in determining TREC and KREC intervals with taking into account the patient’s age and sex are of no small importance for diagnosing IEI in infants.

PIDs are still considered rare diseases, although they are not orphan. In 2019, a total of 2,798 patients with IEI were registered in the Russian Federation, with 60% of them being children [38]. The introduction of programs for mass newborn screening based on an evaluation of the TREC and KREC levels will significantly increase the risk group for SCID and other severe PIDs that cause death at an early age. Early diagnosis will ensure the possibility of timely application of pathogenetically appropriate therapy. This also relates to the use of radical transplantation technologies during the opportunity window before the onset of severe clinical manifestations; this will not only improve the quality of life of patients with this pathology and save their lives, but also reduce the financial and economic costs in the treatment and life support of patients [39, 40, 41, 42, 43, 44, 45].

## CONCLUSION

The results of our studies suggest that the gestational age should be considered as an important factor affecting the TREC and KREC levels. In order to interpret the results of newborn screening, we calculated the reference ranges of these parameters for different gestational groups. This also has the potential to allow us to monitor immune changes in T and B cells during immunotherapy, including monitoring after hematopoietic stem cell transplantation.

Early diagnosis and treatment are important in all IEI variants. The use of the method of quantitative assessment of the T- and B-cell markers TREC and KREC, respectively, has made it possible to develop screening programs for SCID and agammaglobulinemia detection in many countries of the world. However, other PIDs such as immunodeficiency disorders with normal levels of peripheral T and B cells, defects in phagocyte count and function, complement deficiency, and diseases associated with immune dysregulation remain unaddressed. Further search for effective disease markers; development of strategies for cellular, genetic, and functional diagnostics; as well as adaptation of these tolls to mass diagnostic programs for various PIDs are required.

## Abbreviations

TREC: T-cell receptor excision circles
KREC: K-deleting recombination excision circles
TCR: T-cell receptor
BCR: B-cell receptor
PID: primary immunodeficiency
IEI: innate errors of immunity
SCID: severe combined immunodeficiency
DBS: dried blood spot.
